# Live attenuated simian immunodeficiency virus vaccination confers superinfection resistance against macrophage-tropic and neurovirulent wild-type SIV challenge

**DOI:** 10.1099/vir.0.000135

**Published:** 2015-07

**Authors:** Neil Berry, Claire Ham, Jack Alden, Sean Clarke, Richard Stebbings, Jim Stott, Deborah Ferguson, Neil Almond

**Affiliations:** ^1^​ Division of Virology, NIBSC, South Mimms, Potters Bar, Hertfordshire EN6 3QG, UK; ^2^​ Divison of Biotherapeutics, NIBSC, South Mimms, Potters Bar, Hertfordshire EN6 3QG, UK

## Abstract

Vaccination with live attenuated simian immunodeficiency virus (SIV) in non-human primate species provides a means of characterizing the protective processes of retroviral superinfection and may lead to novel advances of human immunodeficiency virus (HIV)/AIDS vaccine design. The minimally attenuated SIVmacC8 vaccine has been demonstrated to elicit early potent protection against pathogenic rechallenge with genetically diverse viral isolates in cynomolgus macaques (*Macaca fascicularis*). In this study, we have characterized further the biological breadth of this vaccine protection by assessing the ability of both the *nef*-disrupted SIVmacC8 and its *nef*-intact counterpart SIVmacJ5 viruses to prevent superinfection with the macrophage/neurotropic SIVmac239/17E-Fr (SIVmac17E-Fr) isolate. Inoculation with either SIVmacC8 or SIVmacJ5 and subsequent detailed characterization of the viral replication kinetics revealed a wide range of virus–host outcomes. Both *nef*-disrupted and *nef*-intact immunizing viruses were able to prevent establishment of SIVmac17E-Fr in peripheral blood and secondary lymphoid tissues. Differences in virus kinetics, indicative of an active process, identified uncontrolled replication in one macaque which although able to prevent SIVmac17E-Fr superinfection led to extensive neuropathological complications. The ability to prevent a biologically heterologous, CD4-independent/CCR5^+^ viral isolate and the macrophage-tropic SIVmac316 strain from establishing infection supports the hypothesis that direct target cell blocking is unlikely to be a central feature of live lentivirus vaccination. These data provide further evidence to demonstrate that inoculation of a live retroviral vaccine can deliver broad spectrum protection against both macrophage-tropic as well as lymphocytotropic viruses. These data add to our knowledge of live attenuated SIV vaccines but further highlight potential safety concerns of vaccinating with a live retrovirus.

## Introduction

Human immunodeficiency virus (HIV), the causative agent of AIDS, infects approximately 35 million people worldwide with an estimated 2.3 million new cases in 2012 (UNAIDS, 2013). Development of a safe, effective and affordable prophylactic AIDS vaccine would have a major impact on global health. However, 30 years after the identification of HIV the most effective responses that a successful vaccine needs to elicit remain uncertain.

Experimental infection of macaques with simian immunodeficiency virus (SIV) is generally considered the most appropriate model for understanding details of HIV pathogenesis and unravelling the scientific principles of prophylactic vaccine protection. Vaccination with live attenuated SIV delivers potent and durable vaccine protection against wild-type challenge (Connor *et al.*, 1998; Daniel *et al.*, 1992; Fukazawa *et al.*, 2012; Koff *et al.*, 2006; Mansfield *et al.*, 2008; Norley *et al.*, 1996). In cynomolgus macaques (CM; *Macaca fascicularis*), prior exposure to the *nef*-disrupted live attenuated virus SIVmac251/C8 (SIVmacC8; Rud *et al.*, 1994) confers potent and durable protection against subsequent infection with wild-type SIV, delivered either as cell-free or cell-associated virus (Almond *et al.*, 1995) and against heterologous as well as homologous viral challenge (Berry *et al.*, 2008, 2011). However, neither serological responses (Almond *et al.*, 1997), nor CD8^+^T-cell-mediated immunity (Stebbings *et al.*, 2004, 2005) appear to identify a central role for adaptive immune responses in this model of vaccine protection. Recent studies have identified strong innate responses to SIVmacC8, particularly localized responses in lymphoid tissues immediately following vaccine delivery, which may impact on the early protection observed in our studies (Ferguson *et al.*, 2014).

Dissecting the relative potency of vaccination of CM with SIVmacC8 has indicated that protection may vary both according to the duration of vaccination, for example, between studies that compare short (3 week) and long (20 week) vaccine regimens and against challenge with genetically homologous and heterologous viruses (Berry *et al.*, 2008, 2011). These studies indicate that genetic and therefore antigenic relatedness are not central to protection, particularly at early times. Crucially, the genetically more closely related challenge virus (i.e. the SIVmac251/32H/L28 reisolate) appeared more capable of bypassing protection compared with the genetically more distinct SIVsmE660 stock. Hence, alternative biological properties may account for differential outcomes of challenge of SIVmacC8-vaccinated macaques with different wild-type virus stocks.

Infection with attenuated SIV causes dynamic and persisting changes to CD4 positive and CD4 negative lymphocyte populations in blood and lymphoid tissues (Li *et al.*, 2011; Manoussaka *et al.*, 2013). Thus, altered host cell tropism of the challenge virus could enable a virus to bypass the protection conferred by an attenuated virus vaccine with a distinct viral tropism. SIVmacC8 is ostensibly T-cell tropic, targeting CCR5^+^/CD4^+^ lymphocytes early in the infection process (Cantó-Nogués *et al.*, 2001; Ferguson *et al.*, 2014) and multiple lymphoid tissues resulting in the establishment of persistent virus infection in a highly dynamic process. During this early critical period, reservoirs of virus infection are established which persist over time. CD4-independence has been reported to lead to macrophage tropism of SIV (Puffer *et al.*, 2002) and perivascular macrophages represent a primary cell type which are able to become productively infected by SIV (Williams *et al.*, 2001), with subsequent consequences of neurological sequelae and neuropathologic effects of SIV in the brain. Hence, we were interested to establish the outcome of challenge with a biologically distinct virus, particularly to challenge with macrophage-tropic strains with reduced CD4-dependence for cell entry *in vitro* and a distinct pattern of cell distribution following infection *in vivo* and whether this would impact on characterized neuropathological outcomes. Characterization of vaccine outcomes in such a scenario has not been reported previously.

In this study we compared the ability of vaccination with SIVmacC8, or its wild-type *nef-*intact counterpart SIVmacJ5, to protect against challenge with macrophage-tropic and/or neurovirulent strains, SIVmac17E-Fr and SIVmac316. The implications of the findings from these studies are presented in the context of understanding the broader issues of vaccine protection delivered by a live retrovirus.

## Results

### Replication kinetics of SIVmac17E-Fr in cynomolgus macaques

**Fig. 1:**
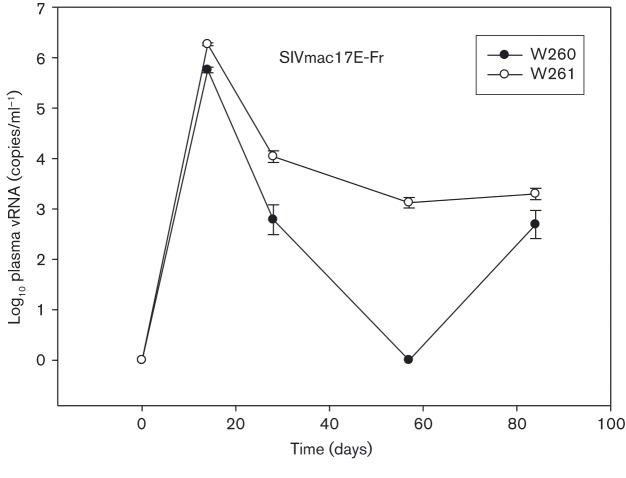
Infectivity of SIVmac17E-Fr in CM. Plasma vRNA profiles of two cynomolgus macaques (W260/W261) to determine the initial infectivity of SIVmac17E-Fr during the acute and immediate post-acute phase.

Initially, to assess the infectivity and replication potential of the SIVmac17E-Fr stock in CM, two naïve subjects (W260/W261) were inoculated intravenously with 50 TCID_50_ SIVmac17E-Fr (Flaherty *et al.*, 1997). Reproducible plasma virus load kinetics were detected (Fig. 1) peaking at day 14 post-infection (p.i) at ∼10^6^ SIV RNA copies (ml plasma)^− 1^, but decreasing rapidly (100–1000 fold) by day 28. At day 84, post SIVmac17E-Fr challenge viral RNA (vRNA) levels were ∼10^3^ SIV RNA copies ml^− 1^. Both macaques seroconverted to SIV Gag and Envelope antigens by 84 days p.i. (data not shown). This preliminary characterization of the properties of SIVmac17E-Fr in CM enabled a vaccine/challenge study to be performed.

### Plasma viral RNA dynamics of SIVmacC8, SIVmacJ5 and SIVmac17E-Fr

**Fig. 2:**
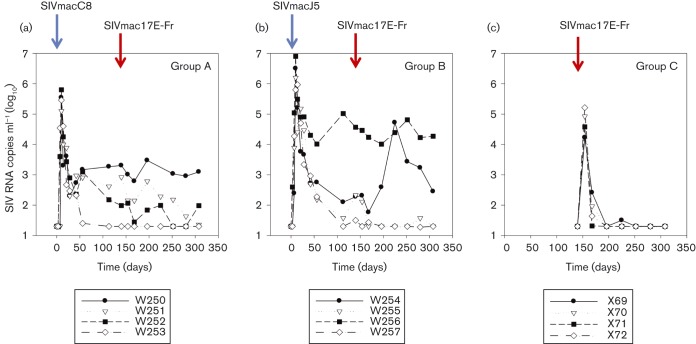
Comparative vRNA analysis of the vaccine/challenge study. Log_10_ SIV RNA copies ml^− 1^ plotted against time (days) for macaques initially immunized with either SIVmacC8 (a, Group A) or SIVmacJ5 (b, Group B) followed by challenge with SIVmac17E-Fr after 140 days, as indicated by the red arrows. Naive controls, (c, Group C) were challenged with SIVmac17E-Fr at the same time.

Viral infection replication dynamics were determined for groups of CM inoculated with SIVmacC8 (W250–W253), SIVmacJ5 (W254–W257) or SIVmac17E-Fr (X69–X72) for 140 days (20 weeks). Individual vRNA levels measured by real-time PCR at frequent intervals during acute infection (Fig. 2a, b) identified differences at both early and later times between SIVmacC8 and SIVmacJ5. Plasma vRNA levels following SIVmacC8 infection peaked at day 10 p.i. (mean log_10_ 5.47 ± 0.14 sem SIV RNA copies ml^− 1^), as expected, with a subsequent control in vRNA load similar in all four macaques to day 42. Thereafter, viral RNA kinetics differed between individuals that exhibited either (i) very effective control of vRNA load (W253; < 50 RNA copies ml^− 1^ at all time points), (ii) poor control (W250; >1000 RNA copies ml^− 1^ at all time points) or (iii) intermediate control (W251, W252 where vRNA levels fluctuated in the 30–1000 RNA copies ml^− 1^ range).

Viral RNA kinetics following wild-type SIVmacJ5 infection also peaked at day 10 p.i., but at a higher level (mean log_10_ 6.35 ± 0.23 sem SIV RNA copies ml^− 1^) with virus loads between days 10 and 42 reduced >1000-fold in all macaques, after which plasma vRNA was controlled, falling in W254, W255 and W257 to < 300 RNA copies ml^− 1^. In marked contrast, however, for macaque W256, plasma vRNA levels remained at a high steady state between 10^4^ and 10^5^ SIV RNA copies ml^− 1^ through to 140 days p.i.

These data compare with the plasma viral load profiles of four naive CM infected with SIVmac17E-Fr (X69–X72), which exhibited distinct kinetics from either SIVmacC8 or SIVmacJ5 (Fig. 2c). Peak plasma vRNA load was detected at 14 days p.i. (mean log_10_ 4.74 ± 0.21 SIV RNA copies ml^− 1^) followed by a rapid drop to the limit of detection in all four macaques by day 56. Plasma vRNA loads were < 50 RNA copies ml^− 1^ in all four macaques for the remaining 120 study days.

### Cell-associated viral load

The kinetics of virus replication in blood detected by plasma vRNA were reflected in peripheral blood mononuclear cell (PBMC) co-culture data with the use of indicator cells. Following infection with SIVmacC8 or SIVmacJ5, mean log_10_ peak cell-associated virus loads at day 10 p.i. were 2.4 ± 0.8 and 3.0 ± 0.7 SIV infected cells per 10^6^ PBMCs, respectively. Following this peak, detection of virus by co-culture became more intermittent in all subjects except W256 where virus was recovered at all times through to 20 weeks p.i. Following infection with SIVmac17E-Fr at day 14 p.i., mean log_10_ virus load was 1.3 ± 0.6 SIV infected cells per 10^6^ PBMCs. At later times, recovery of virus by co-culture with CEMx174 cells became increasingly intermittent and undetectable beyond 12 weeks p.i. (data not shown).

### SIVmacC8 or SIVmacJ5 infection prevents subsequent infection with SIVmac17E-Fr

At day 140 p.i. with SIVmacC8 or SIVmacJ5, CM W250–W257 were inoculated intravenously with 50 TCID_50_ SIVmac17E-Fr along with naive controls X69–X72. Analysis of vRNA levels by quantitative reverse transcriptase (qRT)-PCR demonstrated all four naive controls were infected (Fig. 2c), indicated by a spike in SIVmac17E-Fr plasma RNA levels. A range of molecular markers were used to examine for evidence of breakthrough of SIVmac17E-Fr in macaques inoculated with either SIVmacC8 or SIVmacJ5. Although perturbations in vRNA levels were identified in both SIVmacC8- or SIVmacJ5-inoculated macaques when challenged with SIVmac17E-Fr, no secondary spikes in vRNA were identified attributed to viral superinfection. Indeed, for most there was a decrease in virus load detectable at one or more time-points 140–196 days p.i. However, for W254/SIVmacJ5 a marked (1000-fold) increase in viral load was detected between days 168 and 224 of the study, before declining thereafter.

**Fig. 3:**
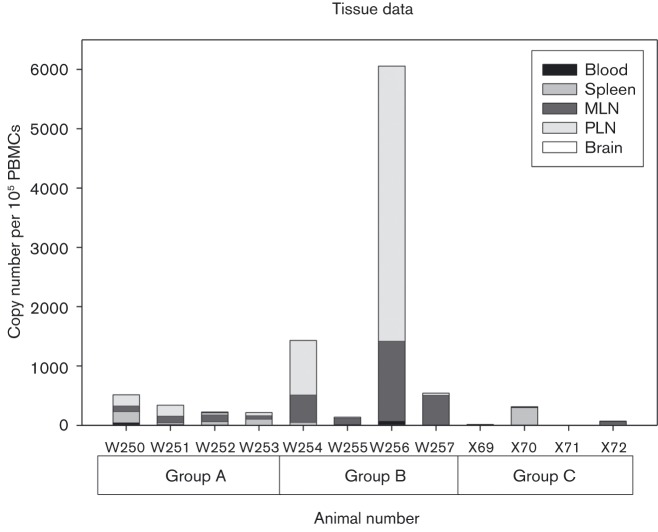
Proviral SIV DNA levels. SIV DNA levels, expressed as copies per 100 000 cells represented as a composite bar graph for each macaque for blood, spleen, mesenteric lymph nodes (MLN), peripheral lymph nodes (PLN) and brain recovered *post-mortem*. Total SIV *gag* levels are represented from groups A and B after 280 days, whereas Group C (SIVmac17E-Fr) was challenged for 140 days.

Analysis of blood, lymphoid tissue and brain samples by sensitive DNA PCR specific for SIV *gag* sequences sequestered at late-stage infection is presented in Fig. 3. Total SIV DNA loads appear to reflect overall plasma virus load detected by RT-PCR, with markedly higher loads detected in W254 and W256. Mesenteric lymph nodes (MLN) and spleen expressed the majority of the SIV DNA signal for each macaque. *Nef*-specific PCR assays employed to identify the presence of SIVmac17E-Fr sequence indicated that in both peripheral blood and tissue analysis there was no evidence of breakthrough of the rechallenge virus in any SIVmacC8- or SIVmacJ5-inoculated macaques (Table 1). Evidence of total SIV *gag* viral RNA was sought in cerebrospinal fluid (CSF) samples *post-mortem* in all macaques by RT-PCR, including controls, though none was detected (data not shown). Earlier samples of CSF were not available for analysis. Neither were we able to detect SIV DNA in brain tissue recovered *post-mortem* in either vaccine groups or SIVmac17E-Fr controls. Overall analysis of the same samples by SIV *nef-*specific discriminatory PCR to differentiate SIVmac17E-Fr from SIVmacC8 or SIVmacJ5 did not detect any evidence of SIVmac17E-Fr in any macaque initially inoculated with either SIVmacC8 or SIVmacJ5.

**Table 1:** Detection of SIVmacC8, SIVmacJ5 or SIVmac17E-Fr by restriction endonuclease digestion analysis in blood and lymphoid tissues at termination

Group	ID	Virus (week 0)	Blood C8/J5 17E-Fr	Spleen C8/J5 17E-Fr	MLN C8/J5 17E-Fr	PLN C8/J5 17E-Fr
A	W250	SIVmacC8	+ −	− −	+ −	+ −
	W251		− −	+ −	+ −	+ −
	W252		− −	+ −	+ −	+ −
	W253		− −	+ −	+ −	+ −
B	W254	SIVmacJ5	+ −	+ −	+ −	+ −
	W255		+ −	+ −	+ −	+ −
	W256		+ −	+ −	+ −	+ −
	W257		+ −	+ −	+ −	+ −
C	X69	–	−	+	+	+
	X71		−	−	+	+
	X72		−	+	+	+
	X73		+	−	+	+

SIVmac17E-Fr was detected in X73 in PBMCs; all controls (X69–X73) signalled SIVmac17E-Fr positive in MLN and peripheral lymph nodes (PLN), but no signals for challenge virus were detected in any macaques immunized with SIVmacC8 or SIVmacJ5. Persisting signals for SIVmacC8 or SIVmacJ5 were detected in all tissue samples, except SIVmacC8/W250/spleen.

### Serology

**Fig. 4:**
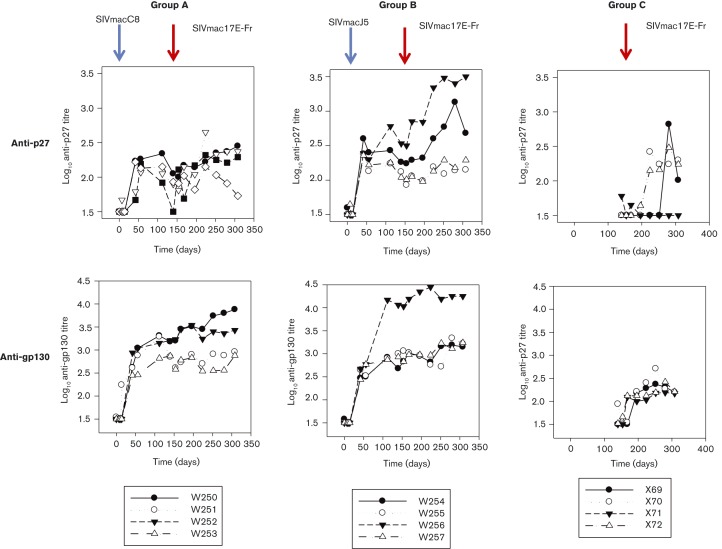
SIV antibody levels. Log_10_ anti-gp130 *env* and anti-p27 *gag* titres for Group A (SIVmacC8, W250–W254), Group B (SIVmacJ5, W254–W257) and challenge controls, Group C (SIVmac17E-Fr, X69–X72).

All macaques seroconverted to SIVmacC8 and SIVmacJ5 with recombinant SIV envelope detected by indirect enzyme immunoassay (EIA) at 6 weeks pi. At 20 weeks p.i., end-point titres ranged between log_10_ 2.5 and 4.0 (Fig. 4). Anti-SIV Gag antibodies tended to be lower and not always sustained to 20 weeks p.i. (Fig. 4). Anti-gp130 and anti-p27 *gag* responses further reflected individual virus replication levels, irrespective of the immunizing virus, although anti-gp130 titres were generally higher than anti-p27. By 4–8 weeks post-SIV exposure, anti-gp130 titres had increased and plateaued (2.5–3.0 log_10_ range), with SIVmacJ5 exhibiting higher titres, as expected. As with the vRNA data, W256 was an outlier in this group exhibiting a substantial rise in antibody titres (>4.0 log_10_) post SIVmac17E-Fr challenge. All naive, unvaccinated macaques challenged with SIVmac17E-Fr mounted an anti-gp130 response, although titres were lower than for either SIVmacC8 or SIVmacJ5; anti-*gag* responses to SIVmac17E-Fr were more variable with X71 failing to make anti-p27 antibodies. Hence the overall ability for SIVmac17E-Fr to stimulate strong humoral responses appears limited. Overall, anti-SIV antibody generation appeared to be related directly to individual virus replication kinetics where high levels of anti-gp130 and anti-p27 were markers for sustained viral replication and generalized immunological stimulation.

### CD4 lymphocyte counts

**Fig. 5:**
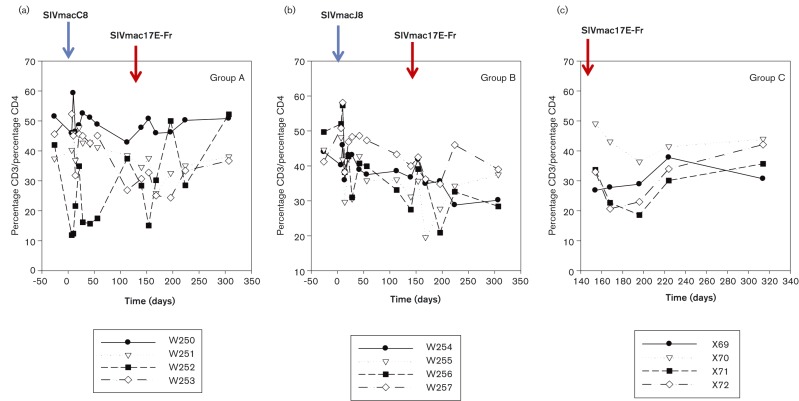
CD4 percentages. Percentage CD3/CD4 levels shown for groups A–C. Groups A and B, percentage CD3/percentage CD4 plotted against time in days, indicating pre-immunization levels (day − 50) and fluctuating levels at time of either SIVmacC8 or SIVmacJ5 inoculation, and subsequently, challenge with SIVmac17E-Fr. Challenge controls, group C (SIVmac17E-Fr) were plotted from time of challenge (indicated) and over the same period as groups A and B.

Amongst macaques infected with SIVmacC8, CD4 lymphocyte counts fluctuated but remained in the 35–55 % range for the entire duration of the study (Fig. 5a). Interestingly, W250, which had the highest persisting SIVmacC8 vRNA levels, maintained the highest, most stable CD4 percentage throughout. Amongst macaques infected with SIVmacJ5 (Fig. 5b), there was a general downwards trend in the proportion of CD4 positive individuals (44.8 ± 3.8 % to 33.5 ± 5.8 %) over time. In particular, W256 exhibited significant CD4 decline associated with high, persisting vRNA levels in plasma. Profiles of macaques infected with SIVmac17E-Fr (Fig. 5c) were generally stable, with no evidence of CD4^+^ lymphocyte loss over the 20 weeks of infection, although there was a transient dip in CD4^+^ lymphocytes immediately post SIVmac17E-Fr inoculation in three out of the four controls.

### Inoculation with SIVmacC8 or SIVmacJ5 prevents SIVmac17E-Fr-related neuropathology

**Fig. 6:**
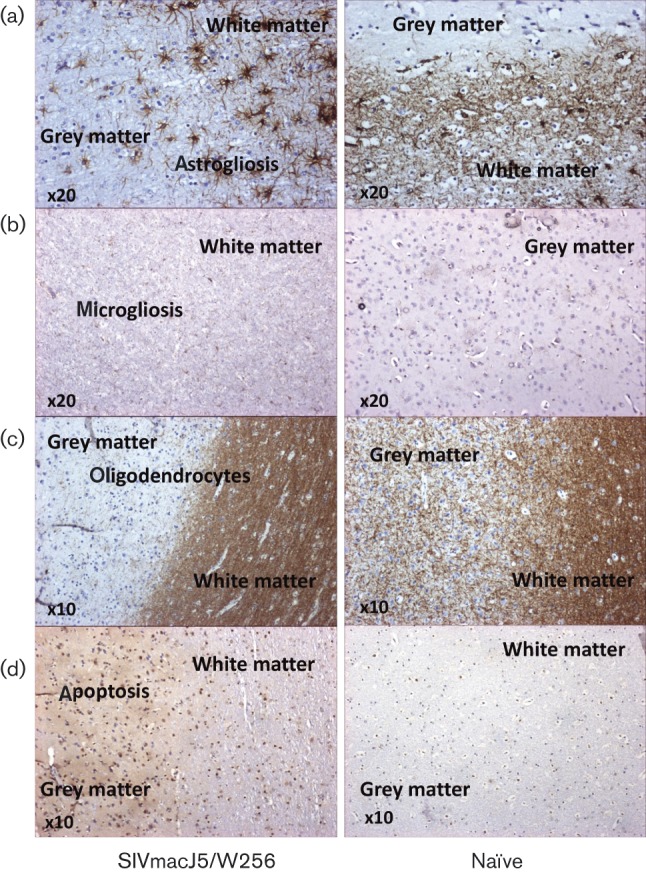
Neuropathology of SIVmacJ5/W256 compared with naive controls. The effects of uncontrolled replication of SIVmacJ5 in macaque W256 on brain pathology by assessment of sections of frontal lobe (white and grey matter) for evidence of (a) astrogliosis (GFAP), (b) microgliosis/microglial staining (Iba-1), (c) oligodendrocytes (CNPase-1) and (d) apoptosis (caspase-3). Sections of SIV-positive brain were compared at the magnifications indicated with comparable SIV-negative sections.

The primary outcome of challenge with SIVmac17E-Fr was prevention of superinfection by virtue of the presence of an actively replicating retrovirus (i.e. SIVmacC8 or SIVmacJ5). However, what appears to be an active process where continued virus replication of the immunizing virus is able to prevent viral superinfection also leads to unwanted side-effects where continual virus replication is deleterious. In the majority of vaccinates, the extensive neuropathology induced by SIVmac17E-Fr in unvaccinated controls as described by Clarke *et al.* (2012) contrasts sharply with the majority of either SIVmacC8 or SIVmacJ5 vaccinates, which were protected from the most severe effects. However, to highlight the differential neurological profile of W256/SIVmacJ5, which exhibited a sustained, uncontrolled profile of virus replication, we analysed sections of frontal lobe for markers for astrogliosis, microgliosis, impaired oligodendrocyte activity and apoptosis (Fig. 6). Specifically, Fig. 6(a) indicates staining of the frontal lobe with glial fibrillary acidic protein (GFAP) highlighting astrocyte activation and astrogliosis in white and grey matter in W256/SIVmacJ5, compared with uninfected control tissue samples. Fig. 6(b) indicates increased staining of the microglial marker Iba-1 compared to naive controls. In oligodendrocytes located in the frontal lobe there was an increased level of CNPase activity, compared with naive controls (Fig. 6c) and raised levels of caspase 3, a marker for apoptosis (Fig. 6d). Taken together, these data indicate that despite protection from the more extensive neuropathology induced by SIVmac17E-Fr there was nevertheless a clearly defined index of pathology associated with uncontrolled replication of SIVmacJ5 which was detrimental to the host.

### Outcome of challenge of SIVmacC8 vaccinates with SIVmac316

**Fig. 7:**
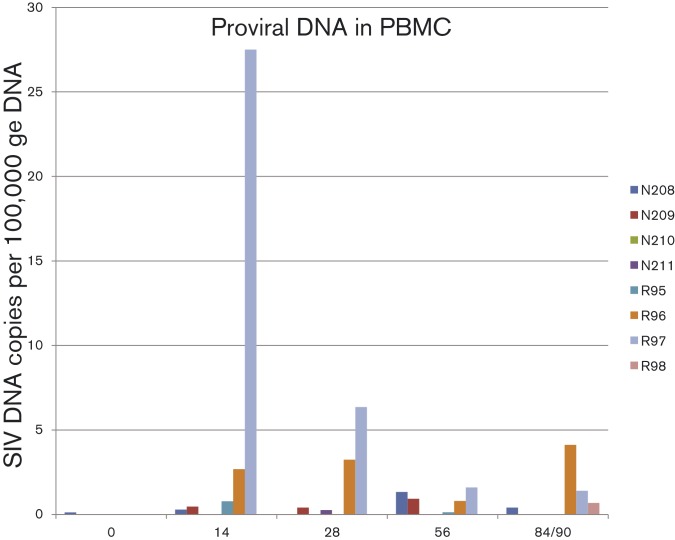
Distribution of SIV DNA in SIVmacC8 vaccinates after challenge with SIVmac316. SIV DNA levels expressed as *gag* DNA copies per 100 000 genome equivalents (ge) in PBMCs during the time-course of infection.

To investigate further the outcome of challenge with alternative macrophage-tropic virus challenges we retrospectively analysed the outcome of a vaccine/challenge study utilizing another derivative of SIVmac239, designated SIVmac316 (Mori *et al.*, 1993, 2000). Four CM (N208–N211) were vaccinated by intravenous inoculation with SIVmacC8, which all seroconverted to gp140 *env* and p27 *gag* as previously described (Silvera *et al.*, 2001). After a period of 76 weeks, macaques were challenged intravenously with 100 MID_50_ SIVmac316. Despite a prolonged period between initial vaccination and challenge, all four vaccinates remained protected against SIVmac316 rechallenge as determined by quantitative DNA PCR in blood (data not shown) and viral burden analysis of tissues *post-mortem* (Fig. 7). Attempts to reisolate virus at termination from peripheral blood, MLN, spleen and peripheral lymph nodes (PLN) were also negative 12 weeks after administration of challenge virus. Although levels were low, SIV DNA was detected in all controls post-challenge and all seroconverted to SIV rgp140 but no anamnestic antibody response in vaccinates was identified (data not shown).

## Discussion

Vaccination with live attenuated SIV can confer potent protection against subsequent exposure to wild-type virus. However, it is extremely challenging to exploit these vaccine observations in humans, because protective efficacy appears to be inversely proportional to the degree of attenuation of the vaccine virus (Johnson *et al.*, 1999) and the mechanisms that contribute to vaccine protection have been difficult to establish. In CM, protection against both homologous and heterologous virus challenge has been described (Berry *et al.*, 2008, 2011), although protection was superior against a genetic and antigenically distinct challenge compared with a homologous challenge that exhibited accelerated replication kinetics. This led us to investigate whether protection with live attenuated virus is associated with other biological properties distinct from the antigenic relatedness between challenge and vaccine viruses. The biological tropism of SIV strains used in previous studies to challenge CM infected with attenuated SIVmacC8 have been predominantly T-cell tropic. By contrast, few data exist relating to protection against macrophage-tropic viruses. Here, we report that vaccination of CM with SIVmacC8 elicits potent protection against detectable superinfection with either of two macrophage-tropic strains derived from SIVmac239: the neurotropic SIVmac17E-Fr (Flaherty et al., 1997) and SIVmac316 (Mori *et al.*, 1993, 2000) isolates. The latter was engineered by introducing envelope sequence changes found in viruses recovered from alveolar macrophages of rhesus macaques infected with SIVmac239. Furthermore, in the case of challenge with SIVmac17E-Fr, we demonstrated that vaccine protection was not dependent upon vaccination with the *nef*-disrupted live attenuated SIVmacC8 but was also possible by vaccination with its *nef-*intact, wild-type homologue SIVmacJ5 which retains an undisrupted *nef* open reading frame.

These data add intriguing information regarding the protection against pathogenic SIV challenge conferred in macaques by prior infection with a replicating retrovirus. Previously, our understanding of this protection was based around the premise that the first replicating virus should express an attenuated phenotype. Clearly, there exists an inverse correlation between the level of attenuation and protection (Johnson *et al.*, 1999). However, the scientific paradigm of protection was based upon the vaccinating virus eliciting adaptive immune responses to prevent superinfection. Thus, it would be counterintuitive to immunize with a wild-type virus that would overtly disrupt the immune system. The outcome of protection by macaques in group B that were previously infected with wild-type SIVmacJ5 does not fit this paradigm. Moreover, since the replication kinetics of macaque W256 in this group appeared uncontrolled, compatible with well-established survival implications for high plasma load if left untreated (Lifson *et al.*, 1997; Staprans *et al.*, 1999; Watson *et al.*, 1997), it might be expected that the host would in fact be more susceptible to superinfection. This was not the case as W256/SIVmacJ5 was still able to prevent superinfection with SIVmac17E-Fr, despite an inability to control its own replication. Moreover, there is evidence to suggest that W256/SIVmacJ5 increased in replication efficiency after SIVmac17E-Fr challenge, which may either be coincidental, or compatible with the immunizing virus exhibiting a boost in replication as previously observed in protective vaccination scenarios (Berry *et al.*, 2008, 2011).

There is an increasing body of evidence indicating that adaptive immunity is not central to the protection obtained, particularly in CM, by prior exposure to attenuated SIV, either because modulating adaptive immunity fails to abrogate protection (Almond *et al.*, 1997; Stebbings *et al.*, 2005), or the breadth of protection does not correlate with genetic and therefore antigenic relatedness (Berry *et al.*, 2008, 2011). If adaptive immunity is not central to protection, or at least only a component part, then other mechanisms need to be explored. This study was designed to investigate whether target cell blocking was critical. T-cell tropic strains, such as SIVmacC8, have a distinct biological phenotype from macrophage-tropic strains and infect a different range of host cells based upon their CD4-dependence and co-receptor usage. If direct infection of target cells was required to prevent a second SIV strain from superinfecting, then rechallenge with a macrophage-tropic virus of a macaque ‘vaccinated’ with a T-cell-tropic attenuated virus would simply bypass this block. The data presented here indicates that this does not seem to be the case.

The neurotropic, neurovirulent SIVmac17E-Fr strain initially characterized extensively in macaque models of HIV (Clements *et al.*, 2002; Edinger *et al.*, 1997; Flaherty *et al.*, 1997; Mankowski *et al.*, 1997) has been demonstrated to lead to infiltration in the brain, where infection of microglial/macrophage-related cells represents a reservoir for the virus in a CD4-independent manner. In CM, we have further shown the ability of this virus to result in extensive neuropathology (Clarke *et al.*, 2012), despite a primary vRNA profile which was both limited and transient (Figs 1 and 2c). Attempts to rescue viral sequence by PCR from CSF samples after this time were, perhaps not surprisingly, unsuccessful, further reflecting a more limited productive infection of SIVmac17E-Fr in this species. Recently, in rhesus macaques, depletion of CD4^+^T-cells prior to SIV infection, which leads to the emergence of CD4-independent SIV-envelopes, where SIV-positive macrophages represented a very high proportion of all SIV RNA positive cells, has been described (Micci *et al.*, 2014). Hence, under the right conditions, SIV appears capable of establishing a persisting infection in macrophages which, if blocks to CCR5^+^T-cells were in place via vaccination, would provide a viable alternative for infection.

However, this would not appear to be the case as both SIVmacC8 and SIVmacJ5 appear to effectively block and prevent SIVmac17E-Fr superinfection. Moreover, these data would appear to support the hypothesis, although not definitively prove, that protection is not cell-specific and that target cell blocking is unlikely to be a central feature of live attenuated SIVmacC8 protection in CM. Unfortunately, we were unable to examine specific evidence for infection in a wider range of different tissues (e.g. the lung), where potential infection of alveolar macrophages by the SIVmac316 isolate, for example, would have been informative. We cannot, therefore, exclude the possibility of infection, perhaps at low levels, in these distal sites.

If target cell blocking is not a central feature of protection, other mechanisms need to be explored. Recently, we have reported that in rhesus macaques infected with a genetically engineered live attenuated SIV variant (SIVrtTA), which replicates in the presence of doxycycline, ‘vaccination’ results in global T-cell population changes that are partially reversible when the doxycycline treatment is removed for 8 weeks (Manoussaka *et al.*, 2013). These global changes, if replicated following infection with SIVmacC8, could create a situation where uninfected CD4^+^ lymphocytes are refractory to infection with a newly introduced SIV strain. Such a mechanism may also account for protection mediated by a wild-type virus which disrupts the immune system. Alternatively, induction of innate antiviral responses following infection with the vaccine virus may deliver a potent antiviral immune state to prevent subsequent viral superinfection. We have recently reported that infection with SIVmacC8 elicits a number of innate responses, initially associated with the interferon response to the primary viraemia and subsequent persisting changes, particularly in macrophage and dendritic cell populations (Ferguson *et al.*, 2014). The impact of significant levels of virus replication elicited by SIVmacC8 during primary infection in multiple tissues seems to be central to the mode of protection conferred by SIVmacC8, stimulating innate responses which are present as early as 3 days p.i., but which persist for prolonged periods. This induction and persistence seems a key feature either having a direct effect, e.g. early protection, or at later times after the maturity of adaptive responses. As the timetable of rechallenge with SIVmac17E-Fr was late after any interferon response gene expression profiles might have subsided, it is difficult to assess these effects directly. However, changes to cellular subpopulations established during early infection appear to impact on the ability to resist challenge with wild-type virus at later times.

Whatever the mechanism of protection, prior exposure to SIVmacC8 or SIVmacJ5 prevented both viral superinfection and the marked neuropathology associated with neurotropic SIVmac17E-Fr, hence providing an independent biological measure for determining the protection conferred against SIVmac17E-Fr. The method of selection used by Clements and colleagues to generate the infectious SIVmac17E-Fr clone provided a strong biological pressure for neurotropism. The pathology of the resulting clone is, as a result, quite distinct from that of SIVmacJ5 or SIVmacC8 in cynomolgus macaques (Clarke *et al.*, 2012). Failure to observe these hallmark neuropathological features supports the virological data that there is no evidence of SIVmac17E-Fr superinfection in macaques previously immunized with either SIVmacC8 or SIVmacJ5. On the other hand, considering the attenuated kinetics of replication exhibited by SIVmacC8, it is surprising that distinctive neuropathology was observed in all macaques, which was greatest in macaque W256 which failed to control SIVmacJ5 (Fig. 6; Clarke *et al.*, 2012). We are investigating whether this neuropathology is specific to these CM infected with the minimally *nef*-disrupted, attenuated SIVmacC8. We have preliminary evidence that neuropathology is evident in most macaques infected with SIVmacC8 and similar neuropathology is observed amongst macaques infected with SIVmac239Δ*nef* and SIVrtTA (unpublished findings). Understanding the timing of neuroinvasion with T-cell-tropic, attenuated viruses and the mechanisms of neuropathogenesis may contribute to the development of improved treatments for HIV-associated neurocognitive disorders.

In summary, our attempt to bypass the potent protection conferred by live attenuated SIVmacC8 in CM by viruses which exhibit macrophage-tropic properties and/or neuroptropism failed. In the process, these observations are compatible with the notion that the protection observed is unlikely to be mediated by direct target cell blocking alone, nor is it a feature confined only to genetically attenuated virus strains. These data add to our understanding of how attenuated SIV confers vaccine protection. The novel features of the protection conferred by this approach mean that, ultimately, it may be possible to harness the protective mechanism to complement vaccines that mediate protection through the stimulation of cognate adaptive immunity.

## Methods

### Experimental outline

Purpose-bred juvenile cynomolgus macaques (*Macaca fascicularis*) were used in all studies and confirmed to be simian D-type retrovirus infection free. All were housed and maintained in accordance with UK Home Office guidelines for the care and maintenance of non-human primates. All macaques were sedated with ketamine hydrochloride before inoculation with virus or venepuncture and killed by overdose of anaesthetic. Four animals were challenged with 5000 TCID_50_ of the 9/90 pool of SIVmac32H/C8 (W250–W253) and four with SIVmac32H/J5 (W254–W257). Venous blood samples taken at frequent intervals during the period of the primary viraemia were as previously described (Clarke *et al.*, 2003). An initial analysis of the infectivity of SIVmac17E-Fr was undertaken with two macaques (W260, W261) challenged with 50 TCID_50_ SIVmac17E-Fr (a kind gift from Dr Janice Clements). On day 140 (20 weeks post SIVmacC8/SIVmacJ5 challenge), all eight animals were challenged with 50 TCID_50_ SIVmac17E-Fr with an additional four macaques (X69–X72) challenged with the same dose of SIVmac17E-Fr to serve as naive challenge controls. All animals were euthanized at 167 days (24 weeks) following challenge with SIVmac17E-Fr and tissue samples of spleen, MLN, PLN and brain tissue were collected *post-mortem*. Cerebrospinal fluid (CSF) was also collected for vRNA analysis at this final time point. Separately, four CM were vaccinated for an extended period with SIVmacC8 for 76 weeks and challenged with 100 MID_50_ SIVmac316 (N208–N211); four additional macaques (R95–R98) acted as SIVmac316 challenge controls. The SIVmac316 isolate was a kind gift from Professor Ronald Desrosiers.

### Serology

Antibody responses were determined by EIA for Env (anti-gp130) and Gag (anti-p27) using serial dilutions of plasma samples heat-inactivated at 56 °C for 1 h. Antigens used to coat the EIA plates were SIV recombinant gp130 (EVA 655, 1 μg ml^− 1^), LacZ/p27 (0.25 μg ml^− 1^) or GST/SIVmacJ5 Nef (ARP668, 1 μg ml^− 1^). Log_10_ end-point titres were determined by linear regression analysis (Lotus 123 software).

### Detection and quantification of SIV viral RNA and proviral DNA

Levels of plasma vRNA and proviral DNA were compared for individual macaques challenged with either SIVmacJ5 or SIVmacC8 using real-time quantitative PCR assays as previously described (Berry *et al.*, 2008). Univariate statistical analyses were performed to assess quantitative differences in vRNA levels between study groups. Levels of viral quantification were 50 SIV RNA copies ml^− 1^ in plasma and 1 copy SIV DNA per 100 000 mononuclear cell equivalents.

### Differentiation of virus sequence by analysis of Nef sequence

Superinfection breakthrough of SIVmac17E-Fr in SIVmacC8- or SIVmacJ5-vaccinated macaques was determined by *nef-*specific PCR and differential restriction endonuclease digestion. Primers spanning the entire *nef* ORF capable of amplifying SIVmacC8, SIVmacJ5 and SIVmac17E-Fr were used to derive nested PCR products. Amplifications were performed with outer primers (forward, 9077–9098, 5′-ATGGGTGGAGCTATTTCCAGGA-3′; reverse, 9847–9868, 5′-TGAGCGAGTTTCCTTCTTGTCA-3′), inner primers (forward, 9376–9396, 5′-ACCAGTGATGCCACGAGTTCC-3′; reverse, 9827–9846, 5′-GCCATGTTAAGAAGGCCTCT-3′) with AmpliTaq Gold. Unique restriction endonuclease *Nsi*I digestion was employed to differentiate between SIVmac17E-Fr (based on SIVmac239 sequence) and SIVmacC8 or SIVmacJ5.

### Immunohistochemical analyses of brain sections

Immunohistochemical analyses were performed on brain tissue with antibodies to detect staining for astrocytes (GFAP), oligodendrocytes (CNPase), microglia (Iba-1) and apoptosis (caspase-3) as previously described (Clarke *et al.*, 2012).
